# The neurodynamic treatment induces biological changes in sensory and motor neurons in vitro

**DOI:** 10.1038/s41598-021-92682-2

**Published:** 2021-06-24

**Authors:** Giacomo Carta, Giovanna Gambarotta, Benedetta Elena Fornasari, Luisa Muratori, Marwa El Soury, Stefano Geuna, Stefania Raimondo, Federica Fregnan

**Affiliations:** 1 Department of Clinical and Biological Sciences, University of Torino, Regione Gonzole 10, 10043 Orbassano, Italy; 2 Neuroscience Institute Cavalieri Ottolenghi (NICO), University of Torino, Regione Gonzole 10, 10043 Orbassano, Italy; 3 ASST Nord Milano, Sesto San Giovanni Hospital, Milan, Italy

**Keywords:** Cell biology, Molecular biology, Neuroscience, Medical research

## Abstract

Nerves are subjected to tensile forces in various paradigms such as injury and regeneration, joint movement, and rehabilitation treatments, as in the case of neurodynamic treatment (NDT). The NDT induces selective uniaxial repeated tension on the nerve and was described to be an effective treatment to reduce pain in patients. Nevertheless, the biological mechanisms activated by the NDT promoting the healing processes of the nerve are yet still unknown. Moreover, a dose–response analysis to define a standard protocol of treatment is unavailable. In this study, we aimed to define in vitro whether NDT protocols could induce selective biological effects on sensory and motor neurons, also investigating the possible involved molecular mechanisms taking a role behind this change*.* The obtained results demonstrate that NDT induced significant dose-dependent changes promoting cell differentiation, neurite outgrowth, and neuron survival, especially in nociceptive neurons. Notably, NDT significantly upregulated PIEZO1 gene expression. A gene that is coding for an ion channel that is expressed both in murine and human sensory neurons and is related to mechanical stimuli transduction and pain suppression. Other genes involved in mechanical allodynia related to neuroinflammation were not modified by NDT. The results of the present study contribute to increase the knowledge behind the biological mechanisms activated in response to NDT and to understand its efficacy in improving nerve regenerational physiological processes and pain reduction.

## Introduction

Every day, nerves are subjected to tensile forces as a consequence of joint movements or rehabilitation treatments after a nerve injury^1^. In many cases, the positive effects of applying tensile forces on nerves are exploited to facilitate the healing process^2–8^. For example, the neurodynamic treatment (NDT), which is a non-pharmacological intervention consisting of a combination of physiological movements that induce selective repeated uniaxial tension on the nerve^2,9,10^, was described to be an effective treatment to reduce pain in drug-resistant sciatica patients^11,12^ since the end of the eighteenth century. Several clinical trials have shown that NDT is an effective treatment to increase nerve functions and reduce pain in patients with neuropathies of the somatic nerves^13–17^. Also, several preclinical studies on murine sciatic neuropathy models have shown that NDT resolves mechanical and thermal allodynia, which are very common and disabling symptoms among neuropathic pain patients^18^. NDT affects the protein expression of Nerve Growth Factor (NGF) in the homologous dorsal root ganglia (DRG) and the spinal cord metameres involved by the nerve lesion^3,19,20^. Although there is a growing understanding of how mechanical forces influence tissues^21,22^, there is quite poor evidence on biological processes involved during this phenomenon on nerves. The recent discoveries on nerve-related neuropathic models have identified the specific gene expression in the DRG and the spinal cord related to neuropathic pain and mechanical allodynia. TLR2 (Toll-like receptor-2) and YAP (Yes-associated protein) are significantly upregulated in the DRG neurons in several neuropathic pain models, and their suppression is linked to the suppression of mechanical allodynia^23–25^. In particular, TLR2, is a receptor mediating the macrophage recognition of ligands from microbes that promote inflammation in the DRG and spinal cord neurons. Several studies have shown that TRL2 depletion induces mechanical allodynia suppression through the signalling pathway of myeloid differentiation factor-88 adaptor protein (MyD88)/nuclear factor kappa-light-chain-enhancer of activated B cells (NF-κB) in DRG cells^26^. YAP is a transcriptional regulator, its overexpression and nuclear accumulation in the DRG and spinal cord neurons, like motor neurons, is associated to neuroinflammation after a nerve injury and neuropathic pain leading to mechanical hypersensitivity in naive animals^24,27^. Also, the nerve injury-dependent mechanical allodynia is suppressed in YAP knock-down rats and using siRNA against YAP in the spinal cord. The expression of c-JUN (a gene involved in nerve degeneration) in the DRG and the spinal cord has been linked to neuropathic pain conditions and neuroinflammation^28,29^. Also, c-Jun in these neurons has been identified as one of the main hub genes in several neuropathic pain conditions and its suppression is linked to pain relief in several neuropathic pain animal models^30–33^. The gene expression in DRG neurons encoding for mechanosensitive ion channels has been shown to affect the perception of mechanical painful stimuli. In particular, TACAN suppression, an ion channel that is co-localized with TRPV1 (transient receptor potential cation channel subfamily V member 1) in non-peptidergic nociceptors in the human and murine DRGs^34–36^, is significantly linked to the suppression of mechanical hyperalgesia induced by local and systemic inflammation^36^. Furthermore, the upregulation in the DRG of PIEZO1, an ion channel that is responsible for the sensation of non-noxious stretch and compression forces, is linked to the mechanical pain suppression in humans and animal models of neuropathic pain^37–39^.

NDT protocols available in literature are extremely heterogeneous concerning the number of repeated stimuli (from three to three series of 60 repetitions)^14,16^, the amount of nerve elongation (from 0.8 to 15% of the total nerve rest length)^40–42^, the elongation speed (from one to five seconds)^6,42^ and the time of stretch duration (from 1 to 30 s)^16,42^. Despite the positive results obtained from clinical and preclinical studies that link NDT-induced pain reduction and allodynia improvement to changes in the peripheral and central nervous system, fundamental aspects to obtain standardized treatment protocols have not yet been defined. To overcome this lack of knowledge, the aim of the present research is threefold: (1) to define whether a single set of repeated mechanical stimuli could induce biological and morphological changes in neurons; (2) to define whether NTD could lead to negative effects on neurons and (3) to investigate whether those changes have a dose-dependent behavior.

In the field of biomedical research in vitro experiments are mandatory to avoid animal use as much as possible^9^. Nevertheless, to obtain in vitro data suitable to be translated in humans, it is fundamental to adopt a biological model with the same features of human cells. Taking into account this aspect, to assess the effects of NDT, we selected a mouse motor neuron-like cell line (NSC-34)^43–46^ and a rat nociceptive sensory neuron-like cell line (50B11)^47,48^ as biological models. Indeed, although not all the biological processes are shared among different species, in our research we focused on genes linked to neuropathic pain and stress mechanisms that are expressed in mice, rats, and humans^35,37,49–51^.

## Results

### Effects of neurodynamic treatment protocols on cell morphology

Morphological analyses were performed to assess specifically the possible beneficial or toxic effects of NDT protocols on cell differentiation, a process that is related to nerve healing and sensitive to environmental stimuli^2,45,47,52–55^. Also, effects on neurite growth, a process that is fundamental for neural repair and target tissue re-innervation^7,45,47^, and neurites orientation^2,56–61^.

**Figure 1:**
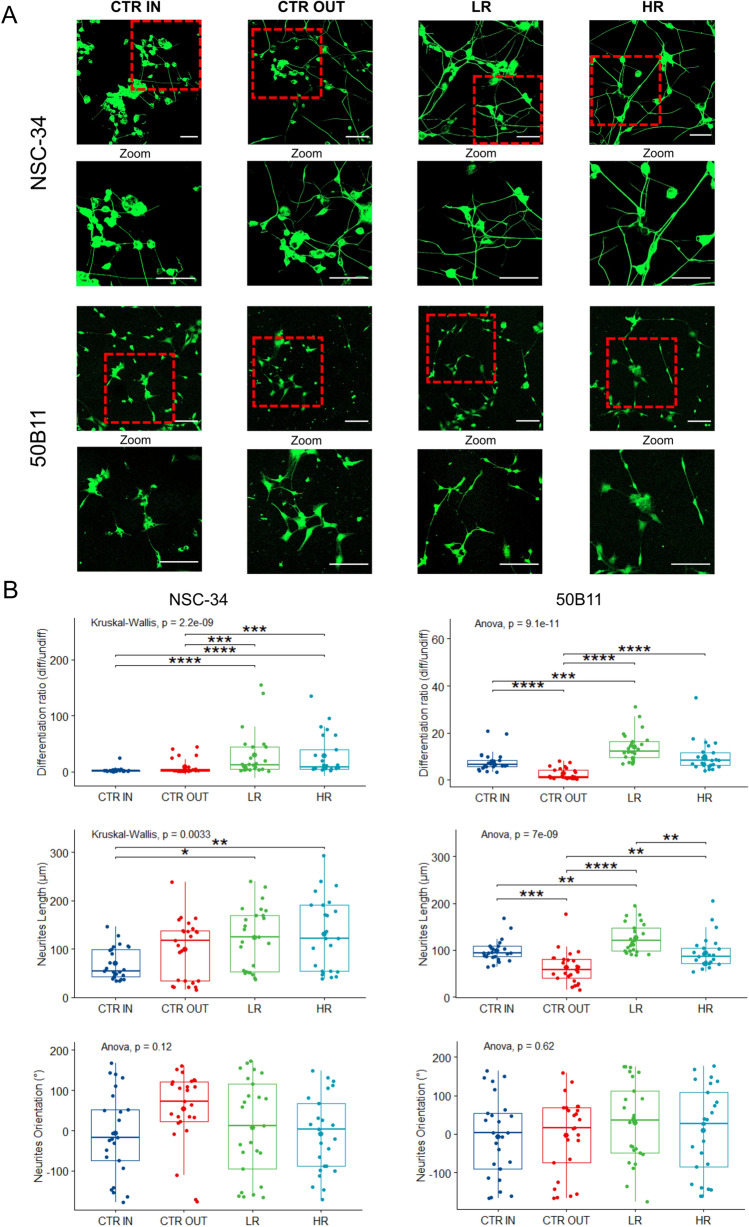
Effects of different neurodynamic treatment protocols on cell morphology. (**A**) Representative images of NSC-34 and 50B11 neurons stained with βIII-tubulin are reported for each type of experimental protocol. Scale bar: 100 µm. Red boxed regions correspond to zoom panels (down), which highlight the cells features following the treatment. (**B**) Quantitative analysis of differentiation ratio, neurites length, and neurites orientation. Values in the graphics are expressed as mean ± SD. For normally distributed data with comparable variances, One-way ANOVA was carried out, while Kruskal–Wallis-test was used for nonparametric data; asterisks show statistically significant differences; *p* ≤ 0.01, ***p ≤ 0.001, and ****p ≤ 0.000.

Representative images of NSC-34 (motor) and 50B11 (sensory) neurons for each type of experimental protocol are reported in Fig. 1A. A significant and dose-dependent response of NDT protocols was detectable for both cell lines (Fig. 1B). The differentiation ratio was significantly higher in NSC-34 cells treated with both LR and HR protocols (Fig. 1B, Table 1 Supplementary for statistical analysis) than in cells belonging to CTR IN or CTR OUT groups. In particular, the median value of the differentiation ratio was 12.00 points (95% CI: 5.25–25) in the LR protocol, 8.6 points (95% CI: 5.5–30) in the HR protocol, and 2.11 points in the CTR OUT protocol (95% CI: 1.33–4). The CTR IN protocol differentiation ratio of 2.00 points (95% CI: 1.47–2) was not statistically different from the CTR OUT. Similar results were detected in 50B11 cells with a significantly higher differentiation rate in LR and HR protocols compared to CTR IN and CTR OUT groups (Fig. 1B, Table 1 Supplementary for statistical analysis). Notably, the differentiation ratio of the LR group was significantly higher than the CTR OUT protocols of 11.33 points and of 7.29 points in the HR protocol. Also, the CTR IN protocol has shown a differentiation ratio significantly higher than the CTR OUT protocol of about 5.19 points.

No significant differences were detected on neurite length for all experimental groups compared to CTR OUT in motor neurons (Fig. 1B, Table 1 Supplementary for statistical analysis). However, a significantly higher neurite length of about 60 µm was detected for the LR and HR protocols compared to the CTR IN group. Also, the sensory neurons treated with LR and HR protocols had significantly longer neurites when compared to those observed in the CTR OUT protocol. The neurites were significantly longer than those of the CTR OUT protocol by about 63.03 μm in the LR protocol and by about 31.28 μm in the HR protocol. Interestingly, the neurites length of the CTR IN protocol was significantly greater than that of the CTR OUT protocol of about 35.62 μm. No significant differences were detected between all protocols on neurites orientation for motor and sensory neurons (Table 1 Supplementary for statistical analysis). In an experimental design where the neuronal models received two different NDT protocols where the CTR OUT represents the sham group, the results showed that both NDT treatments induce a significant increase in cell differentiation and neurites outgrowth, while neurite orientation is not affected.

### Effects of neurodynamic treatment protocols on cell apoptosis

**Figure 2:**
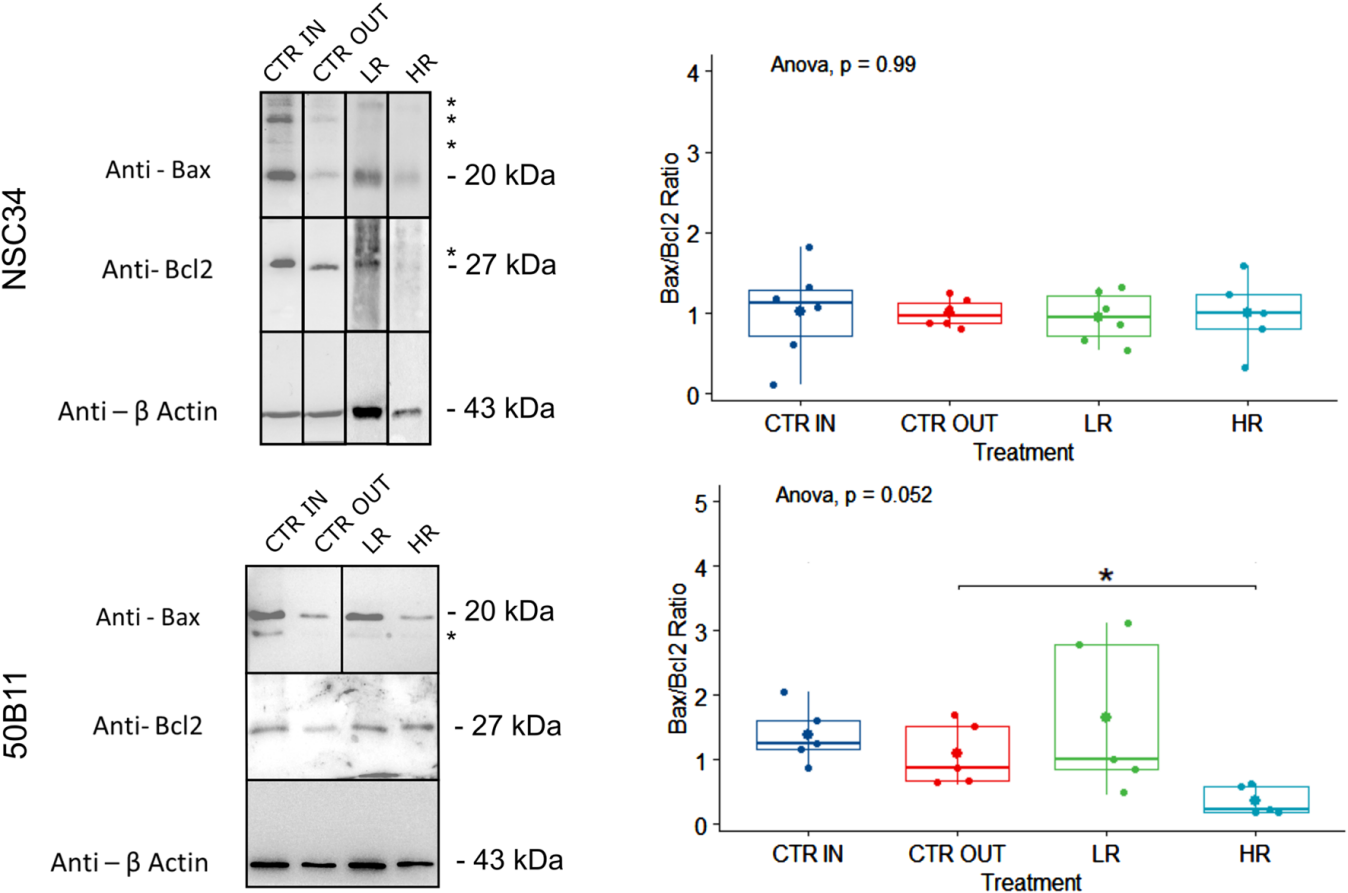
Effects of different neurodynamic treatment protocols on protein expression. Protein expression analysis for Bax and Bcl2 markers on untreated neuronal cells (CTR IN), cells positioned in the bioreactor but untreated (CTR OUT), and cells treated with low (LR) and high (HR) repetitions of neurodynamic treatment. Experiments were carried out in biological quintuplicate (n = 5). On the left, representative western blots are shown; actin was used as a loading control. Upper panels correspond to NSC-34 cells; lower panels correspond to 50B11 cells. Asterisks (*) identify unspecific bands. The samples derive from the same experiment and gels/blots were processed in parallel. Full-length blots and gels are presented in Supplementary Figs. 1–2. In the right panels, the quantitative analysis of all samples is shown. Values in the graphics are expressed as mean ± SD. One-way ANOVA was carried out (data are normally distributed with comparable variances); asterisk shows the statistically significant difference (*p ≤ 0.05).

Proteins were analyzed to assess specifically the possible beneficial or deleterious effects of NDT protocols on neuron survival and death. In particular, the expression of Bax (a pro-apoptotic protein) and Bcl2 (an anti-apoptotic protein) was quantified. A not-pro-apoptotic behavior was induced on sensory and motor neurons by NDT protocols showing the absence of negative effects on cell survival (Fig. 2). Surprisingly, the Bax and Bcl-2 ratio revealed relevant changes in the survival cell profile induced by NDT protocols: a dose-dependent response and anti-apoptotic behavior was found for 50B11 cells with a significant effect for HR protocol compared to CTR OUT (F[3, 20] = 3.18; *p* < 0.05; 95% CI: − 1.21 to 0.54; η_p_^2^ = 0.374, large effect). No significant difference was detectable between the CTR IN and the CTR OUT protocols (F[3, 20] = 0.68; *p* = 0.902; 95% CI: − 0.96 to 1.56; η_p_^2^ = 0.374, large effect). No significant anti-apoptotic effects were detected for NDT and CTR IN protocols on NSC-34 cells (F[3, 20] = 0.03; *p* = 0.992; η_p_^2^ = 0.005, small effect).

### Effects of neurodynamic treatment protocols on gene expression

**Figure 3:**
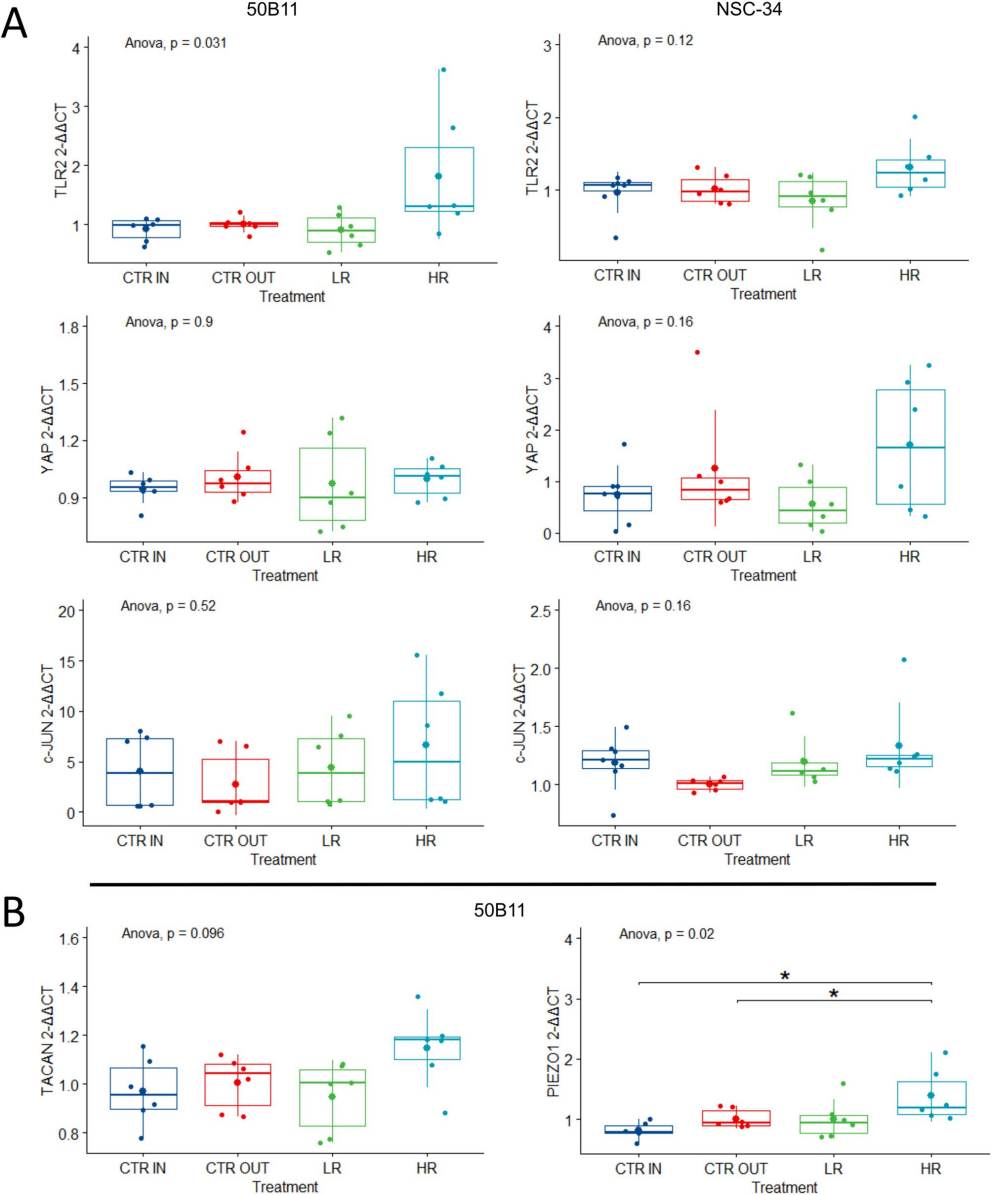
Quantitative gene expression analysis of mechanical allodynia and neuropathic pain markers. Relative quantification (2^−ΔΔCt^) of genes was evaluated by qRT-PCR to assess benefits or side effects induced by neurodynamic protocols on sensory and motor neurons. TATA-binding protein (TBP) was used as a housekeeping gene to normalize data. All data were calibrated to CTR OUT samples. Values in the graphics are expressed as mean ± SD. One-way ANOVA was carried out (data are normally distributed with comparable variances). (**A**) Expression of TLR2, YAP, and c-JUN is reported to assess the effects of NDT on mechanical allodynia and neuropathic pain. No statistically significant differences with CTR OUT were observed. (**B**) Expression of TACAN and PIEZO1 is reported to assess the effects of NDT on mechanical hyperalgesia and mechanical anti-nociception in sensory neurons 50B11; asterisks show statistically significant differences (*p ≤ 0.05).

Gene expression linked to immune response mediated mechanical allodynia (TLR2) and neuropathic pain (YAP, c-JUN) was evaluated in sensory and motor neurons after two different NDT protocols (HR and LR; Fig. 3A). Differently, gene expression linked to mechanical hyperalgesia (TACAN) and mechanical stimuli detection (PIEZO1) was evaluated only in sensory neurons (50B11) since those ion channels play a key role in the sensory stimuli transduction from the periphery to the central nervous system (Fig. 3B).

No significant difference between all experimental groups was detectable for TLR2 expression in sensory neurons (50B11) (F[3, 20] = 3.61; *p* < 0.03 ; η_p_^2^ = 0.351, large effect) and motor neurons (NSC-34) (F[3, 20] = 1.90; *p* = 0.161; η_p_^2^ = 0.214, large effect). Moreover, significant differences of YAP expression were not detectable between the different protocols in both sensory (F[3, 20] = 0.37; *p* = 0.77; η_p_^2^ = 0.053, small effect) and motor neurons (F[3, 20] = 1.92; *p* = 0.158; η_p_^2^ = 0.214, large effect).

The c-JUN expression revealed no significant differences between all experimental groups in both 50B11 and NSC-34 (respectively F[3, 20] = 0.77; *p* = 0.524; η_p_^2^ = 0.103, small effect; and F[3, 20] = 0.77; *p* = 0.12; η_p_^2^ = 0.237, large effect). Since TACAN and PIEZO1 ion channel functions are related to sensory stimuli transduction, only 50B11 cell line was assessed for the expression of those genes.

The expression of ion channels linked to mechanical hyperalgesia (TACAN) revealed no significant differences, among all protocols (F[3, 20] = 2.42; *p* = 0.098; η_p_^2^ = 0.267, large effect).

Interestingly, a significant dose- dependent behavior was detected between the HR and CTR IN protocols (F[3, 20] = 4.18; *p* = 0.013; η_p_^2^ = 0.382, large effect) and between HR and CTR OUT protocols for PIEZO1 expression (F[3, 20] = 4.11; *p* = 0.019; η_p_^2^ = 0.381, large effect).

To summarize, the gene expression results showed no significant changes induced by NDT on genes involved in neuroinflammation and neuropathic pain (TLR2, YAP, c-JUN) in both motor and sensory nociceptive neurons revealing no effects in promoting allodynia or mechanical pain. A significant positive effect was detected for the HR protocol in the sensory nociceptive neurons with an upregulation of PIEZO1, a low threshold mechanosensitive ion channel, responsible for mechanical pain suppression and non-noxious mechanical stimuli transduction.

## Discussion

In this study, two different dosages of NDT were tested on sensory and motor neurons to deepen the knowledge of the biological mechanisms activated in response to repeated tensile forces administration.

To our knowledge, this is the first in vitro model of NDT using motor and sensory neuron cell lines and it shows that a treatment, commonly used in rehabilitation programs to relieve pain and reduce disability in patients, promotes neuroplasticity and regenerative processes selectively on neurons without any detectable negative effect.

Higgins and colleagues have previously demonstrated using a human neuroblastoma cell line (SH-SY5Y), that seven days of continuous progressive mechanical stimuli promote differentiation and neurite outgrowth similar to NGF administration^2^. Unlike other experiments, in this study, we have shown that a single session of mechanical stimuli (NDT) is sufficient to affect the cell differentiation in both sensory and motor neurons. We have also determined that neurite outgrowth is strongly promoted more in sensory than in motor neurons by both NDT tested protocols. As regards the orientation of regenerating axons, we hypothesized that the induced uniaxial tension with our bioreactor could influence the orientation of axonal growth leading to fiber alignment. Indeed, several in vitro studies have shown that continuous linear mechanical stretch can guide the neurites to follow the direction of the administered force^2,58,59^. In our study, both in sensory and motor neurons, the neurites orientation was not affected by the uniaxial tension created by the NDT. This was probably due to the fact that our protocols were administered once, and not for days or weeks as was performed in other studies, and that the stretch was intermittent and not progressive. To better understand the biological effects of mechanical stimuli on neurons, we analyzed proteins linked to cell survival and genes linked to mechanical allodynia and hyperalgesia that have been described in neuropathic pain models.

The cell survival analysis performed, evaluating the ratio between Bax (a pro-apoptotic protein) and Bcl2 (an anti-apoptotic protein) expression, demonstrated that 30 repetitions of mechanical stimuli (HR protocol) had a significant anti-apoptotic effect in sensory nociceptive neurons, promoting cell wellbeing.

Based on the in vivo experimental results on neuropathic pain models treated with NDT^3,6,7,18,20^, a pain modulation effect was described in particular on mechanical pain and mechanical allodynia. Since allodynia is linked to an upregulation of YAP, TLR2, or c-JUN^23–25,28,29,62^ (genes linked to neuroinflammation and immune response) in the DRG and spinal cord, we assessed in motor and sensory neurons their expression profiles.

The lack of significant differences between the experimental protocols has shown that the NDT protocols were not able to promote pro-allodynic responses mediated by the activation of TLR2, YAP, and c-Jun. Otherwise, the analysis performed on sensory nociceptive neurons, derived from rat DRG (50B11), revealed a beneficial effect of NDT in terms of upregulation of PIEZO1 due to HR protocol administration, in accordance with data reported in previous studies that have shown that the PIEZO1 mechanism can suppress mechanical pain^37–39^. Moreover, our data also suggest that NDT does not promote mechanical allodynia as it does not modulate TACAN expression, which is mainly involved in inflammation-mediated mechanical pain^35,36^. Its worth mentioning that all these molecules and their mechanisms are common in rodents and humans^37–39,49–51^ giving our results a high translatability to clinical conditions.

In summary, we have for the first time assessed the effects of NDT protocol dosages on neuronal cells showing positive dose-dependent response linked to morphology, protein, and gene expression levels. Considering that our experiments were not performed on neuropathic pain models, our observations related to the upregulation of the PIEZO1 gene, can only lead us to hypothesize a specific pathway activated by mechanical stimuli for pain modulation. Indeed, further future studies on ex vivo and in vivo neuropathic pain models will be essential to confirm if this is the key-pathway that NDT activates to induce a pain modulation effect.

## Methods

### Bioreactor description

**Figure 4:**
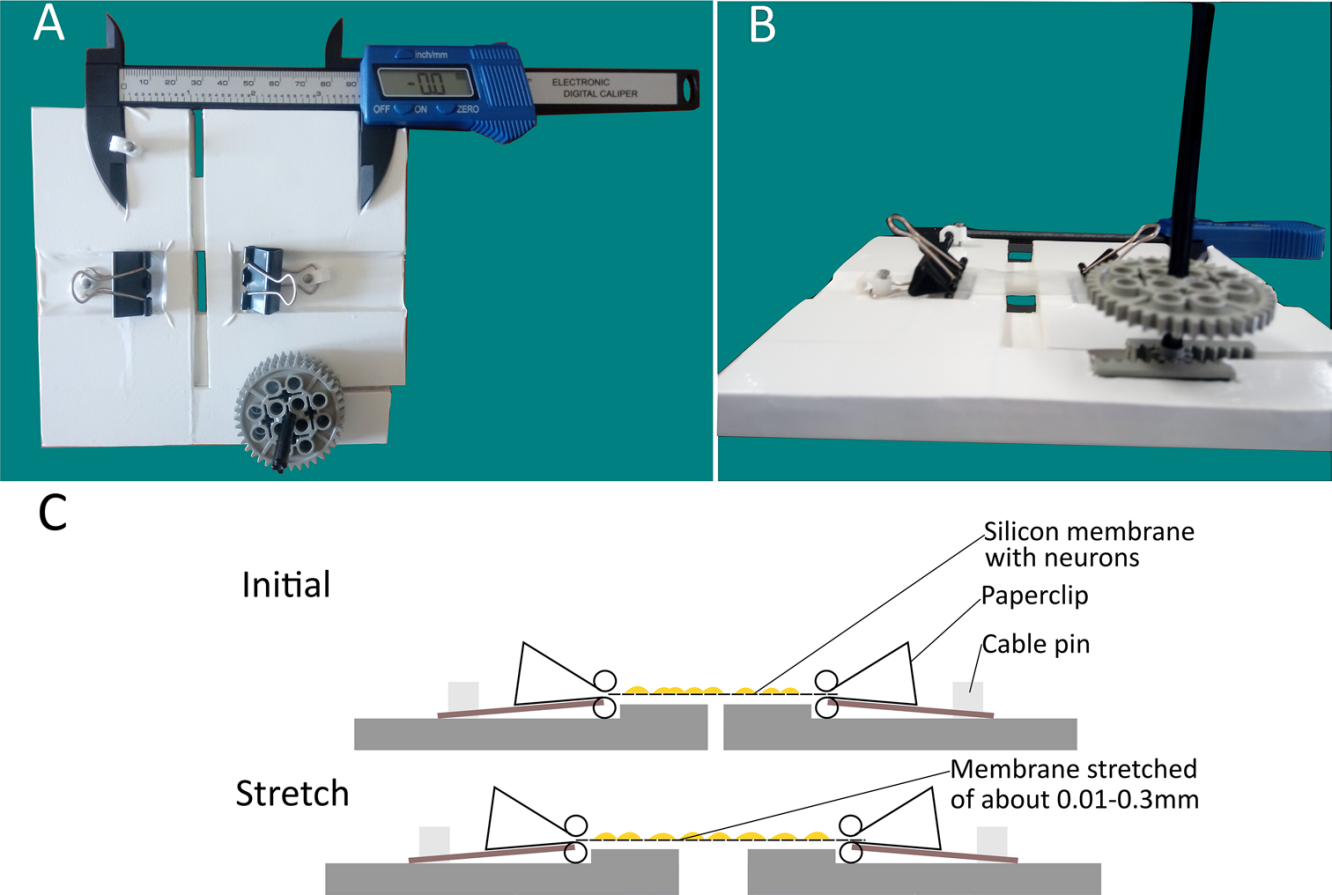
The Bioreactor for in vitro Neurodynamic treatment model. (**A**) The device consists of three 3 mm Forex layers covered with UV-resistant D-c-fix® and divided into two parts puzzled possibly to be driven horizontally by the user to induce neurodynamic treatment protocols. (**B**) Type I collagen pre-coated silicon membrane cut in rectangular shape is clipped into the device and the stretch is administered by hand when turning the wheel to the right. (**C**) The device and the membrane are shown in cross-section. Initially, the membrane is positioned in the device and clipped. To induce the stretch, the wheel is turned to the right, stretching uniaxially the membranes with constant gravity force. Stretch is repeated by turning the wheel to the left until the initial position is reached and turned again to the right with different repetitions depending on the treatment protocols.

As suggested by Sherman and colleagues, a nerve stretch model requires a tool to obtain a controllable, repeatable, mechanical insult^63^. Therefore, to have a reasonable translational power, since the NDT is performed by the assessor’s hands, we had to consider the manual administration of the mechanical stimuli as a fundamental feature of our bioreactor. Since to our knowledge, no bioreactors available on the market can produce uniaxial repeated stretch by hand, we built a bioreactor ad hoc (Fig. 4A, B). A detailed description of the bioreactor can be found in Table 1. Bioreactor sterilization was performed under a cell culture hood by an ethanol 99% wash and with a UV lamp for 30 min.

**Table 1:** Detailed features of the bioreactor used for mechanical stimulation.

**Mechanical and physical features of the bioreactor**
Speed and strain of the uniaxial stretch are regulated by hand with negligible shear stresses on other planes
Gravity is kept constant
Suitable to be used under a class II cell culture hoods
Materials are compatible with 99% ethanol washes and UV sterilization process
Small dimensions are useful to handle the device (9.7 mm high, 140 mm large and 134.5 mm long), to reduce the possible risk of contamination, and to avoid the waste of materials
Possibility of visually monitoring the amount of membrane elongation (electronic caliber)
**Materials commercially available to build the bioreactor**
A triple layer of 3 mm *FOREX* (expanded PVC) for the core structure
UV resistant plastic sheet 0.09 mm thick *D-C-FIX* for the cover
LEGO wheels (one 8 teeth; three 24 teeth) and bars (five 100 mm bars) to produce the bioreactor movements
Two paperclips to keep the silicon membranes attached to the bioreactor
Three plastic cable pins with stainless steel nails covered in nickel to keep the clips and the caliber jointly liable with the bioreactor core
A carbon fiber composite digital thickness gauge with an accuracy of ± 0.1 mm/ ± 0.004 was added to the bioreactor for real-time visual feedback of the elongation induced by the user

### Cell culture

Neuroblastoma × spinal cord cells (NSC-34) were grown in Dulbecco's Modified Eagle's Medium (DMEM, containing 4.5 g/l glucose) with the addition of 10% heat-inactivated fetal bovine serum (FBS; INVITROGEN), 1% (4 mM) l-glutamine, 1% (0.1 mg/ml) streptomycin, and 100 U/ml penicillin^64^. The differentiation medium consisted of DMEM-F12 supplemented with 4 mM l-glutamine, 0.1 mg/ml streptomycin, 100 U/ml penicillin, 1% FBS, and 1 μM retinoic acid.

Immortalized nociceptive sensory neuronal cell line obtained from dorsal root ganglia (50B11) were grown in neurobasal medium (LIFE TECHNOLOGIS, GIBCO) supplemented with 10% FBS (INVITROGEN), 2% B27 (Life Technologies), 0.22% glucose and 0.2 mM glutamine^65^. The differentiation medium consisted of Neurobasal medium added with 50 μM forskolin. Where not specified, reagents were provided by Sigma-Aldrich.

NSC-34 and 50B11 cells were seeded on type I collagen pre-coated silicon membranes (FLEXCELL)^2^ at a density of 15×10^3^ cells per cm^2^. The medium was changed into a differentiation medium two days after seeding the culture. The differentiation medium was changed every two days.

NSC-34 cells differentiate after 2 days and maintain their differentiation state for 8 days^45^ while 50B11 cells differentiate after 2 days and maintain their differentiation state for 4 days^47,48^.

### Cell stretch model parameters

To assess the effects of NDT, the parameters of mechanical stimuli administration were standardized. The silicon membrane elongation was defined as “strain” and the amount of membrane elongation reported as a percentage value was defined as “strain rate”. The strain rate was standardized and refined starting from parameters described in literature. In particular, Clark and colleagues have described from in vivo experiments that the optimal nerve elongation promoting nerve regeneration must be less than the 12% of the nerve length, between 6 and 9%^66^. Nevertheless, silicon membranes compared to nerves have no elastic reserve, meaning that mechanical stimuli applied to the membranes are directly transmitted to the membranes of the neurons seeded on it. Indeed, the stretch force reached when a resistance was perceived by the bioreactor user was quantified using a mean value of the strain induced and was determined to be between 28 and 283 Pa. Those values are lower than the mean strain resistance of the cell membrane of motor neurons (500 Pa) and of sensory neurons (3000 Pa)^67–69^. As a matter of fact, during the NDT treatment, the tissue resistance perceived by the clinician, while the test maneuvers are performed, is used as a standardized hallmark to start the treatment^16,70–72^. For this reason, the amount of the strain rate was set to a range of 0.1–1% of the distance between the two clips of the bioreactor in which the membrane was inserted (31.7 mm; see Fig. 4C). This amount of strain corresponded to the measured onset of the resistance to a strain of the membranes, clipped in the bioreactor, perceivable by the bioreactor user. Notably, the strain rate adopted was between 0.02 and 0.5% of the membrane length, which as described by Rivera & Shah, is the normal mechanical resistance range of peripheral nerves^1^ Moreover, a higher amount of speed, elongation, and duration of the stretch in preliminary trials induced toxic effects leading to massive cell detachment in 10–30 min after the administration of the protocol. The dosage and speed for the Low Repetitions (LR) protocol were set as described by Wang and colleagues since the parameters were fitting with the criteria reported above and they were also effective as a neurodynamic protocol in preventing muscle atrophy after sciatic nerve injury in rabbits^6^. To study a possible dose–response behaviour on neurons a High Repetitions (HR) protocol was performed, fitting with the same parameters described above, but threefold more intense. Both these protocols could be also suitable for future ex vivo and in vivo studies on neuropathic pain mechanisms. An estimated sample of 7 membranes from each group of treatment (effect size f = 0.90; Power [1 − β err prob] = 0.95; α = 0.05; Actual power = 0.97) was evaluated.

### Treatment

NSC-34 and 50B11 cells seeded on silicon membranes pre-coated with type I collagen (FLEXCELL)^2^, were incubated 48 h in differentiation medium; then, they were moved into the bioreactor and treated with the protocols reported below and then returned into the differentiation medium till the end of the experiment (two and five days after the procedure, for respectively 50B11 and NSC-34 cells).

A Low Repetitions (LR) protocol of NDT was administered to the membranes as described by Wang and colleagues^6^, with a cycle of 1 s of stretch (strain rate of about 0.1 and 1% of the membrane rest length), with 5 s to return at the starting position for 10 times. A High Repetitions (HR) protocol followed the same stretch parameters of the LR, but 30 repetitions were administered.

To assess the environmental effects of nourishment privation^57^, induced by the treatment protocols reported above, a sham control, in which membranes were taken off from the medium and positioned in the bioreactor for 90 s (calculated as the mean amount of time between LR and HR protocols in which the membranes were left out of the medium), without the application of mechanical stimuli, was included in the experiment. This experimental group was named “control out” (CTR OUT).

Finally, it was defined the “control in” (CTR IN) protocol, in which membranes were left in the differentiation medium for all the duration of the experiment; the comparison with CTR OUT protocol allowed to assess the effect of taking the membranes out off the medium. Positive effects were defined as those changes linked to nerve regeneration or pain suppression like cell survival, neurite outgrowth, and modulation of genes linked to neuropathic pain and mechanical allodynia. On the opposite, negative effects were defined as those changes induced by the NDT and linked to apoptosis and regulation of the genes linked to pain promotion.

### Immunofluorescent imaging

After 5 and 2 days of culture for NSC-34 and 50B11 respectively, cells were fixed in 4% PFA for 15 min, washed in 0.1 M phosphate buffer (pH 7.2), and processed for immunofluorescence analysis. Samples were permeabilized and blocked in 0.1% Triton X-100, 10% normal goat serum (NGS, VECTOR LABORATORIES INC.), 0.02% NaN3 in Dulbecco's phosphate-buffered saline (PBS; without Ca +  + and Mg + +) for 1 h and incubated overnight with anti-βIII-tubulin (mouse, monoclonal, 1:1000, SIGMA-ALDRICH) primary antibody in PBS; then, after 3 washes at room temperature (RT) in 0.1 M phosphate buffer (pH 7.2), the secondary antibody goat anti-mouse Alexa fluor 488 (1:200, MOLECULAR PROBEC) in PBS was incubated for 1 h at RT. Cells were mounted with a Dako fluorescence mounting medium, the long edges of the membranes were mounted parallel to the long edge of the coverslips. Images were acquired using a Zeiss LSM800 confocal laser microscopy system (ZEISS, Jena, Germany) in a 40 × magnification.

An estimated sample of 25 images, in which all cells were assessed, from each group of treatment (effect size f = 0.40; Power [1 − β err prob] = 0.95; α = 0.05; Actual power = 0.95) was analyzed. The images were randomly acquired from the central portion of the silicon membranes, from a blinded assessor. From confocal cell images, measurements were performed by ImageJ software.

### Differentiation ratio, neurite length, and orientation

The undifferentiated and differentiated cells were manually counted following the criteria described by Maier and colleagues^45^, considering that differentiated cells are characterized by having more than two neurites with at least one of them demonstrating a length that is at least two folds longer than the smallest cell diameter; values were calculated by the ratio between numbers of differentiated and undifferentiated cells for each image. Neurite lengths and orientations were manually measured in all cells as described by Song and colleagues^56^, starting from the soma to the end of each neurite. Only the longest neurite of each differentiated neuron was acquired and the orientation of a neurite was defined as the angle relative to the stretch force direction and the neurite outgrowth^56^.

### RNA isolation, cDNA preparation, and quantitative real-time PCR

RNA extraction, retro-transcription, and quantitative real-time PCR (qRT-PCR) were performed as previously described^73^, using 0.75 µg RNA/sample for retro-transcription. The average value of CTR OUT ΔCt was used as a Ct calibrator for relative quantification. Data normalization was performed adopting TBP (TATA box binding protein) as a housekeeping gene as described in our previous research^74^.

The assessed genes are reported in Table 2; in sensory neurons 50B11 only PIEZO1 and TACAN expression was assessed to detect any effect of NDT on mechanical pain suppression or promotion.

**Table 2:** Sequences of primers used for quantitative real-time PCR and antibodies used for western blot analysis.

Gene	Sequence	Amplicon length (bp)	Accession number	
TLR2	Forward: 5′-CAAACTGGAGACTCTGGAAGCAGG-3′ Reverse: 5′-CACACAGGTAGCTGTCTGCC-3′	125	NM_198769.2 NM_011905.3	
YAP	Forward: 5′-CTTCCTGATGGATGGGAGCAAGC-3′ Reverse: 5′-CTGGTTCATGGCAAAACGAGGGTC-3′	120	NM_001034002.2 NM_001171147.1	
c-JUN	Forward: 5′-ACGACCTTCTACGACGATGCCC-3′ Reverse: 5′- GGGTCGGCCAGGTTCAAGG-3′	116	NM_010591.2 NM_021835.3	
PIEZO1	Forward: 5′-ACTCCTGGCCGGCCTCCC-3′ Reverse: 5′-AGGCGACCTGTGTGACCTGG-3′	122	NM_001077200	
TACAN	Forward: 5′-TGCAGCAGGACTTCCAAGGTATCC-3′ Reverse: 5′-CGCTTCTTCTGGCGTGTGATAGAG-3′	115	NM_001010945.1	

Antibodies for Western blot analysis				
	Code	Dilution	Host	Source
**Primary antibodies**				
actin	A5316	1:4000	Mouse	Sigma
Bax	SC-23959	1:600	Rabbit	Santa Cruz Biotechnology Inc
BcL2	SC-492	1:200	Rabbit	Santa Cruz Biotechnology Inc
**Secondary antibodies**				
HRP conjugated-anti-rabbit	7074	1:15,000	Goat	Cell Signaling
HRP conjugated-anti-mouse	7076	1:15,000	Goat	Cell Signaling

### Western blot analysis

Following RNA extraction, total proteins were extracted with TRIzol Reagent according to the manufacturer's instructions (INVITROGEN, THERMOFISHER). The Laemli buffer (2.5% sodium dodecyl sulfate, 0.125 M Tris-HCl, pH 6.8) at 100 °C was used to dissolve the protein pellet. To determine the protein concentration, the Bicinchoninic Acid assay kit (SIGMA) was used where equal protein quantities (50 μg) were loaded into each lane after being resolved by 12% SDS-PAGE. Western blot analysis was carried out as previously described^75^. Table 2 reports the list of primary and secondary antibodies.

### Statistical analysis

Statistical analyses were performed using R Statistical Software 2020 version (Foundation for Statistical Computing, Vienna, Austria)^76^. The analyses were performed using the car^77^ and rstatix^78^ packages, and plots reported in the manuscript figures were performed using the ggpbur package^79^.

If the normality of the variables assessed was observed (Levene test), One Way Analysis Of Variance (ANOVA) was adopted to assess differences between normally distributed continuous variables with Tukey post-hoc correction. Not normally distributed variables were assessed using the Dunn Kruskal–Wallis multiple comparisons with Bonferroni post-hoc method. The effect size was estimated adopting the partial eta squared (η_p_^2^; 0.010 = small effect; 0.059 = medium effect and 0.138 = large effect)^80^ and for non-normally distributed variables, otherwise epsilon squared (ε_p_^2^) was used (effects: small < 0.08, medium 0.08–0.26, large > 0.26). Confidence Intervals (CI) at 95% were calculated and a statistical significance level of 0.05 was implemented throughout. Values were expressed as mean ± SD (standard deviation). The level of significance was set at *p* ≤ 0.05 (*), *p* ≤ 0.01 (**), *p* ≤ 0.001 (***), and *p* ≤ 0.0000 (****).

## Supplementary Information

Supplementary Information.

## Data Availability

The datasets used and/or analysed during the current study are available from the corresponding author on reasonable request.
