# Supplementary material for: The neurodynamic treatment induces biological changes in sensory and motor neurons in vitro
Source: Sci Rep. 2021 Jun 24;11:13277. doi: 10.1038/s41598-021-92682-2 (PMC8225768; doi:10.1038/s41598-021-92682-2)
Supplement: Supplementary file 1 — Supplementary Information. [file 41598_2021_92682_MOESM1_ESM.pdf]

Supplementary materials

Table 1 Supplementary. Morphological characteristics

NSC-34	CTR IN	CTR OUT	LR	HR	95% CI	Effect size	p
Differentiation ratio (diff/undiff)	2.74 ±4.56	8.69 ±13.6	29.0 ±40.9	28.3 ±36	0.30 – 0.59	ε_p^2=0.437	0.0000
Neurites Length (µm)	69.6 ±33.1	99.3 ±62.2	123 ± 64.2	130 ±74.7	0.14 – 0.06	ε_p^2= 0.437	0.003
Neurites Orientation (°)	-7.26 ±104	54.0 ±92.6	6.76 ±122	-8.75 ±95.0	-48.53 – 34.01	η_p^2=0.059	0.12
**50B11**							
Differentiation ratio (diff/undiff)	7.81 ±4.24	2.63 ±2.35	14.0 ±6.16	9.92 ±6.46	0.44 – 0.71	ε_p^2=0.57	0.0000
Neurites Length (µm)	98.6 ±23.3	63.0 ±34.9	126 ±30.8	94.2 ±35.3	0.26 – 0.58	ε_p^2=0.411	0.0000
Neurites Orientation (°)	-7.68 ±106	-4.40 ±101	28.5 ±105	8.71 ±115	-50.04 – 34.69	η_p^2=0.018	0.62

Values are reported as means ± Standard Deviation (SD). CTR IN, Control group; CTR OUT, sham treatment group; LR, low repetitions neurodynamic protocol; HR, high repetitions neurodynamic protocol; CI, Confidence Intervals ;One-way ANOVA was carried out when data are normally distributed with comparable variances otherwise Kruskal-Wallis was adopted; P, level of significance (P < 0.05).

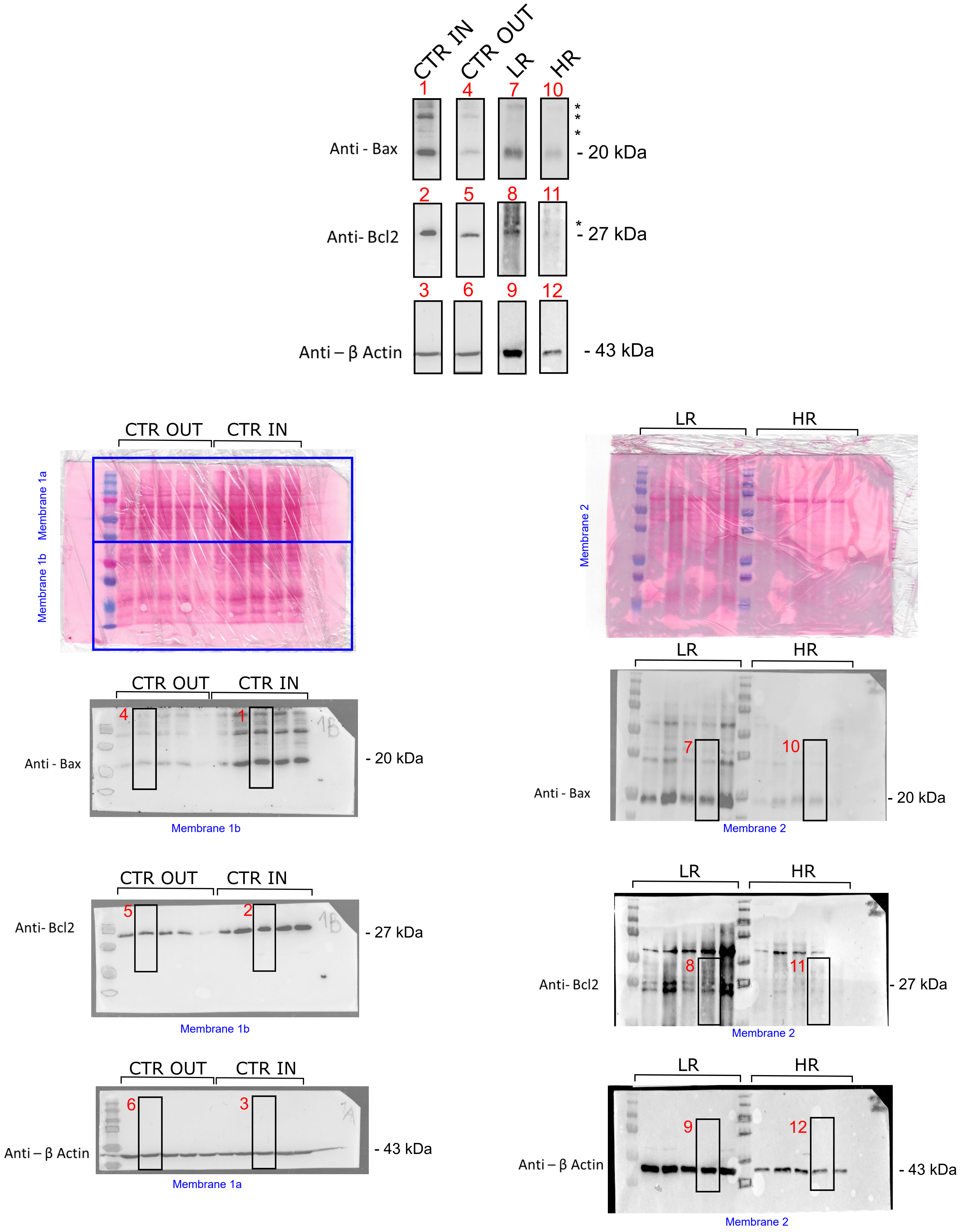

Figure S1. Gels and Blots of NSC34 cells from figure 2- Chemiluminescence detection was performed with Clarity Western ECL Substrate (BIORAD)

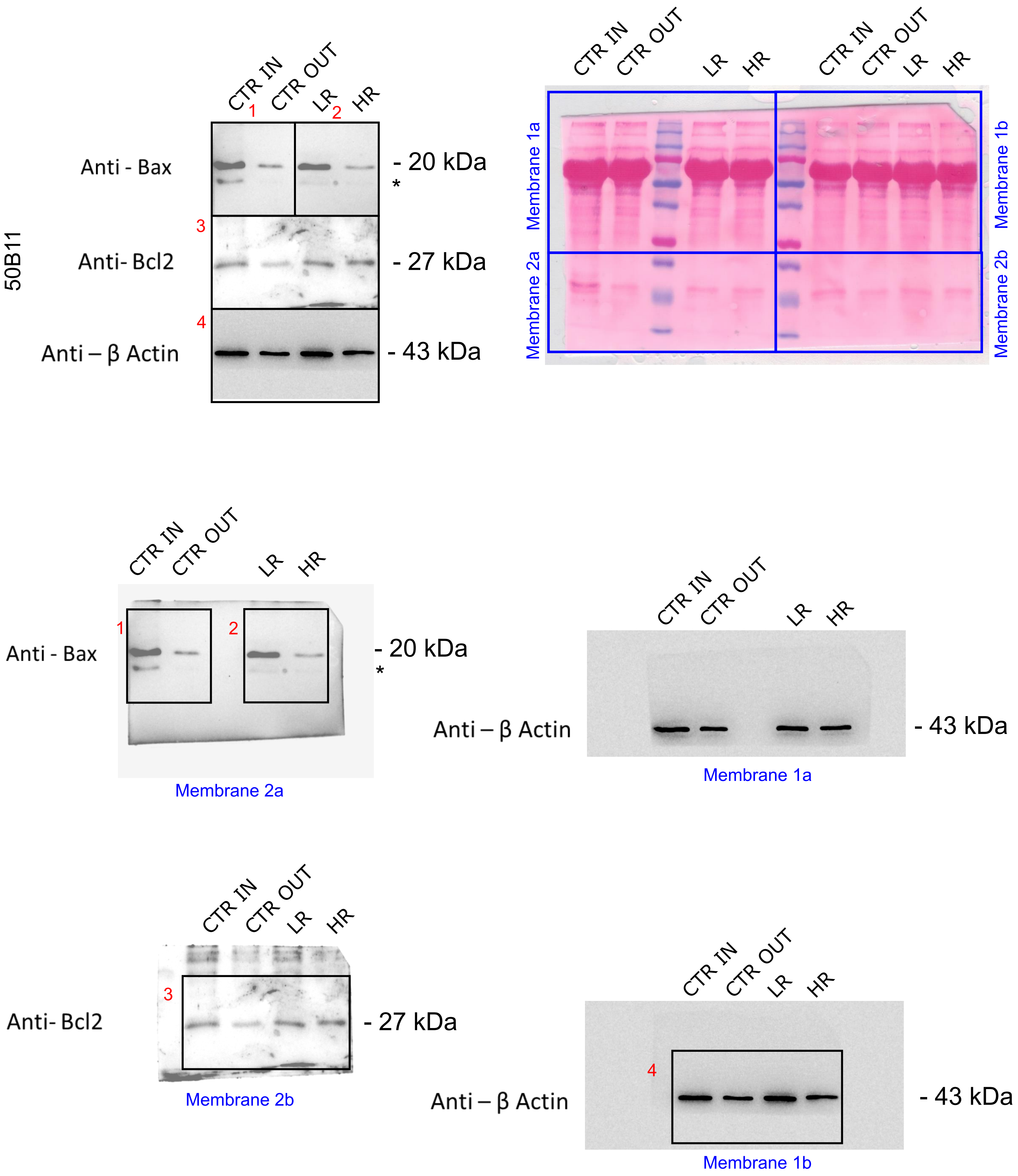

Figure S2. Gels and Blots of 50B11 cells from figure 2- Chemiluminescence detection was performed with Clarity Western ECL Substrate (BIORAD)
